# Hyper IgE Syndrome Associated With Warts: A First Case of Dedicator of Cytokinesis 8 Deficiency in the Philippines

**DOI:** 10.3389/fped.2020.604725

**Published:** 2020-10-30

**Authors:** Jose Carlo Miguel M. Villanueva, Koon-Wing Chan, Remedios C. Ong, Agnes G. Andaya, Yu-Lung Lau, Menno C. van Zelm, Hirokazu Kanegane

**Affiliations:** ^1^Section of Allergy and Clinical Immunology, Department of Pediatrics, University of Santo Tomas Hospital, Manila, Philippines; ^2^Department of Paediatrics and Adolescent Medicine, Li Ka Shing Faculty of Medicine, The University of Hong Kong, Hong Kong, China; ^3^Department of Immunology and Pathology, Monash University and Alfred Hospital, Melbourne, VIC, Australia; ^4^Department of Child Health and Development, Graduate School of Medical and Dental Sciences, Tokyo Medical and Dental University (TMDU), Tokyo, Japan

**Keywords:** dedicator of cytokinesis 8, hyper IgE syndrome, severe atopic dermatitis, T-cell deficiency, warts

## Abstract

Hyper IgE syndrome (HIES) encompasses a group of primary immunodeficiency diseases (PIDs) that is characterized by severe atopy, and recurrent infections and markedly elevated serum IgE levels. The majority of HIES cases suffer from autosomal dominant mutations in the *signal transducer and activator of transcription 3* gene. A minority of cases display autosomal recessive inheritance, and one form is caused by mutations in the *dedicator of cytokinesis 8* (*DOCK8*) gene. Here we describe the first recognized and diagnosed case of DOCK8 deficiency in the Philippines. A 14 year-old-girl was referred due to recalcitrant atopic dermatitis, recurrent sinopulmonary infections, with widespread warts on the face, trunk and extremities. She had no coarse facial features or retained primary teeth, whereas she presented with widespread viral skin infections and multiple allergic diseases. Laboratory examinations revealed elevations in eosinophil count and serum IgE. The level of T-cell receptor excision circles was undetectable. The patient was suspected to have HIES with a probable DOCK8 deficiency. Genetic analysis disclosed a large genomic deletion involving exons 2-4 in the *DOCK8* gene. A combination of recalcitrant atopic dermatitis, asthma, food allergies, with viral skin infections should increase the physician's consideration of a PID. Patients with HIES accompanied by warts and T-cell deficiency can be strongly suspected to have DOCK8 deficiency.

## Introduction

Primary immunodeficiencies (PIDs) are a heterogeneous group of inherited disorders characterized by poor or absent function in one or more components of the immune system (1). PID usually presents as recurrent infections in children, and they are among the most frequent clinical dilemmas for primary care physicians. Aside from recurrent infections, PIDs may present with numerous other clinical features that may cause them to be misdiagnosed and undetected by most clinicians. Hyper IgE syndrome (HIES) is heterogenous syndromic form of PID that presents with severe dermatitis and recurrent infections, with a characteristic markedly elevated serum IgE level (2, 3). The majority of HIES cases are caused by autosomal dominant (AD) mutations in the *signal transducer and activator of transcription 3* (*STAT3*) gene (4, 5); however, a minority of patients suffer from an autosomal recessive (AR) form. Mutations in the *dedicator of cytokinesis 8* (*DOCK8*) gene lead to combined immunodeficiency or AR-HIES (6, 7). DOCK8 is a member of the DOCK family of atypical guanine-nucleotide exchange factors that promote activation of the Ras homolog gene family of small guanine triphosphatase binding protein cell division cycle 42 (CDC42) (8, 9). CDC42 plays an important role in biological processes that involve cytoskeletal rearrangement. Therefore, DOCK8 deficiency results in defective CDC42 activation and subsequently affects cell migration, survival, and effector functions. If not recognized promptly and treated early, the prognosis of the patients is poor, as most do not survive past adolescence (10). Hematopoietic stem cell transplantation (HSCT) represents the only curative treatment (11). Here, we describe the first recognized and diagnosed case of DOCK8 deficiency in the Philippines.

## Case Report

A 14-year-old Filipina girl of Chinese descent was referred to our hospital due to severe dermatitis with warty lesions. The patient was born to a 34-year-old primigravida, and the mother and the father are first-degree cousins, hence she was born from a consanguineous union (Supplementary Figure 1). Her younger brother who had severe molluscum infection died of brain abscess at 5 years old. She presented with dry erythematous skin and pruritic scales from 3rd day of life, and was diagnosed with atopic dermatitis. At 5 years old, the skin continued to be thickened and lichenified over the entire body. This was associated with food allergy to egg, milk, peanut, chocolate, shellfish, and fish. The patient developed pulmonary tuberculosis, which was successfully treated with quadruple anti-Koch's therapy including isoniazid, rifampicin, pyrazinamide, and ethambutol for 6 months. At 7 years old, the patient developed multiple furuncles, with abscesses, impetigo, and cellulitis. Administration of antibiotics improved the symptoms; however, she developed multiple skin-colored papulonodular skin lesions with central umbilication on the entire skin (Supplementary Figure 2A). She was diagnosed with systemic molluscum contagiosum. Within 1 year of follow-up, the patient showed persistent atopic dermatitis, and developed opacification of the left eye, accompanied by dimming of her vision (Supplementary Figure 2B). Yellowish purulent discharges were noted from both eyes. Her condition was complicated by severe atopic dermatitis, persistent allergic rhinitis, asthma, suspected multiple food and drug allergies, recurrent sinopulmonary infections, recurrent skin and soft tissue abscesses, mucocutaneous fungal lesions, and extensive giant molluscum contagiosum lesions. Therefore, the patient was suspected to have an underlying PID.

The patient was stunted but did not show signs of wasting. She did not have dysmorphic facial features or retained primary teeth, but had an oral thrush. She also had a symmetric chest expansion, with rhonchi, coarse crackles, and wheezes over both lungs. Tender, fluctuant, erythematous abscesses were observed on the scalp, as well as in some areas of the trunk.

Laboratory tests revealed normal counts of blood neutrophils and lymphocytes, but eosinophil count was elevated with 1,176 cells/μL (Supplementary Table 1). Serum IgG and IgA levels were 1,841 and 181 mg/dL, respectively, whereas IgM level was low (10 mg/dL, normal range: 50–350). Intriguingly, serum IgE level was extremely elevated (>5,000 kU/L). Although kappa-deleting recombination excision circles were normal, T-cell receptor excision circles (TRECs) were undetectable, indicating an impaired T-cell function (12). Chest X-ray showed no pneumatoceles, and the bacterial cultures of samples collected from eye and skin wound were positive for *Staphylococcus aureus*.

Based on the elevated IgE and eosinophil levels, the patient was suspected to have HIES. According to the National Institute of Health (NIH) scoring system for HIES (13), the patient scored 49, which indicated a probable HIES (>40) (Supplementary Table 2). However, this score may also be suggestive of AD-HIES. The patient, however, did not have any retained primary teeth, scoliosis, or a characteristic face; features that were frequently observed in patients with STAT3-deficient AD-HIES. To clinically distinguish between DOCK8-deficient HIES and AD-HIES, the DOCK8 scoring system was applied (Supplementary Table 3) (14). The patient scored 111.08 (cut off: <30), which strongly suggested a DOCK8 defect, which is a form of T-cell deficiency.

**Figure 1:**
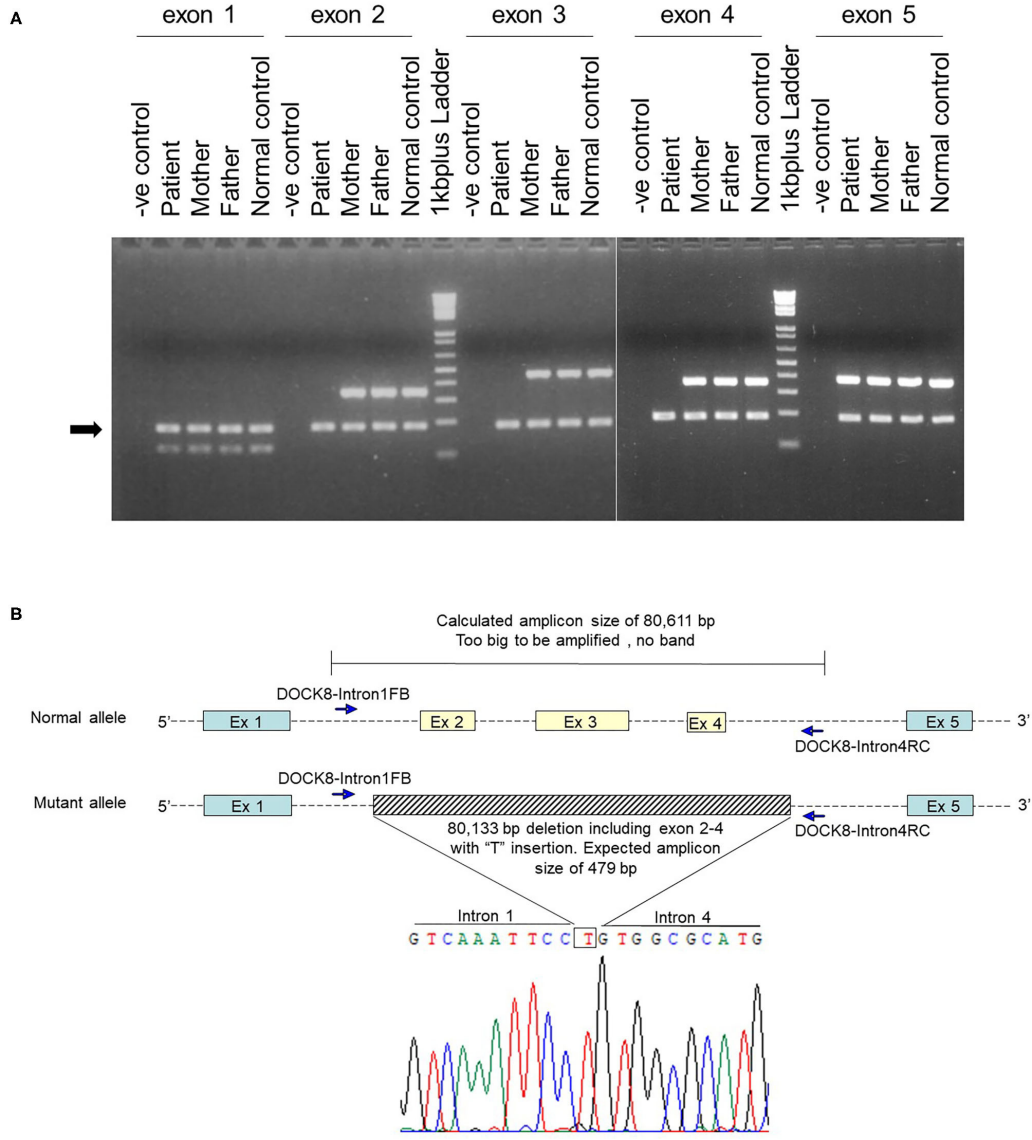
Genetic analysis of a large homozygous *DOCK8* gene deletion. **(A)** Multiplex PCR on genomics DNA was performed by co-amplification of *DOCK8* exon 1 to exon 5 with a reference gene (*CFTR* exon 4). Electrophoresis of PCR products in 3% agarose gel indicated the absence of *DOCK8* exons 2–4 in the patient. The arrow indicates the PCR product of the reference gene. **(B)** After several rounds of PCR, primers located in intron 1 (*DOCK8*-Intron1FB, 5′-GCAGCATTGGCAAAGTGTTCTG-3′) and intron 4 (*DOCK8*-Intron4RC, 5′-CACACTTACAGATGTGTGCTTG-3′) successfully amplified the breakpoint region. Sequencing of this PCR product revealed a deletion of 80,133 bp with an insertion of “T.” The indel mutation started from chr9:218156 to 298288 (GRCh38.p12), *DOCK8* intron 1 position +3,126 to intron 4 position −6,292 (IVS1+3126_IVS4-6292delinsT).

**Figure 2:**
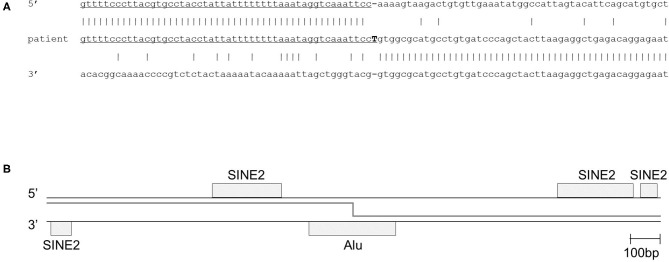
Deletion breakpoint of the patient. **(A)** Sequence of the 5′ breakpoint region, the 3′ breakpoint region and the breakpoint fusion sequence in the patient. **(B)** Analysis of repeat elements in the 2,000 bp flanking each of the breakpoint regions showed short interspersed elements (Alu and SINE2). The 3′ breakpoint is located in an Alu sequence, and the 5′ breakpoint is not located, but closely adjacent to a SINE2 element. SINE, short interspersed nuclear element.

After obtaining a written informed consent from the parents, genetic analysis was performed. Whole-exome sequencing identified a homozygous large deletion of exons 2–4 in the *DOCK8* gene, which was confirmed by multiplex polymerase chain reaction (PCR) on genomic DNA for *DOCK8* coding regions (Figure 1A). PCR with a forward primer in intron 1 and reverse primer in intron 4 amplified the breakpoint regions. Sanger sequencing of this product revealed a deletion of 80,133 bp with an insertion of “T” (Figure 1B). The 3′ breakpoint is located in an Alu transposable element, but the 5′ breakpoint is not located in any transposable elements (Figure 2) (15). Both parents were heterozygous for the same allele with the large deletion, indicating that they were obligate carriers (Supplementary Figure 3).

The patient had recurrent abscesses, persistent pruritus, and extensive molluscum contagiosum lesions despite the good adherence to the treatment regimen instituted. The result of the exome sequencing bolstered the need for HSCT for the patient to survive. Unfortunately, she died of severe sepsis at the age of 14 years.

## Discussion

The majority of HIES represent AD-HIES caused by dominant-negative (DN) mutation in the *STAT3* gene. AD-HIES is also infrequently observed in patients with haploinsufficiency in *ERBB2IP, TGFBR1, TGFBR2* or DN mutations in *CARD11* (1). Recently, 8 kindreds with AD-HIES due to DN *IL6ST* mutations were reported (16). A minority of HIES patients suffer from an AR form, of which DOCK8 deficiency is the most common cause, followed by *IL6R, IL6ST, ZNF341, SPINK5*, and *PGM3* mutations (1). Although DOCK8-deficient patients clinically present as HIES, the disease is classified as a combined immunodeficiency by the International Union of Immunological Societies Expert Committee, because of the profound functional T-cell deficiency (1).

More than 200 documented cases of DOCK8 deficiency have been reported worldwide (10). Its incidence is the highest among people with Turkish and Arabic descent, wherein consanguinity rates are high; however, there has been no report from the Philippines.

DOCK8 deficiency profoundly impairs T helper (Th)17 cell differentiation (17). Th17 effector cells are responsible for recruiting and activating neutrophils important for the host defense against extracellular bacteria and fungi. When dendritic cells encounter these organisms, interleukin (IL)-1 and IL-6 are produced by these cells, and transforming growth factor-β are produced by various cells, which activate the transcription factors retinoic acid-related orphan receptor γt and STAT3, and thus stimulate the differentiation of naive CD4^+^ T cells to Th17 subset (18, 19). However, naive CD4^+^ T cells fail to differentiate into Th17 cells in DOCK8-deficient patients. Decreased Th17 cells, which cause a reduction in IL-17 and IL-22 production, lead to increased susceptibility to extracellular bacterial and fungal infection. Of note, it is speculated that the absence of Th17 cells in HIES leads to unopposed activity of STAT6, thereby increasing Th2 cells, and consequently producing IL-4, -5, and -13, and these cytokines drive the robust elevation of serum IgE and eosinophils (17).

DOCK8 deficiency manifests as a combined immunodeficiency characterized by eczema, recurrent respiratory tract and persistent skin viral infections and markedly elevated serum IgE level. There is also a predisposition toward the development of other allergic diseases including severe eczema, food allergy, allergic rhinitis and severe asthma. Fungal infections range from mucocutaneous candidiasis to invasive disease. There is also an increased risk of developing cancers, in particular, viral-driven cancers such as squamous cell carcinomas due to papilloma virus, Epstein-Barr virus-driven smooth muscle tumors and lymphomas (20). HSCT represents the only curative treatment. If not treated, the prognosis tends to be poor, with only a 37% survival rate by the age of 30 years (21). This is primarily due to life-threatening infections, malignancies, or cerebral events, resulting in early demise.

Our patient had a homozygous gross deletion in *DOCK8*, a type of genetic lesion that is a relatively frequent cause of DOCK 8 deficiency (14). However, for previously described DOCK8-deficient patients, the breakpoint regions have not been determined. Previously, gross deletions in *IGHM, BTK*, and *DCLRE1C* (encoding Artemis) were shown to be associated with transposable elements (22). One breakpoint in our patient was indeed located in an Alu element. However, the other breakpoint was not, and the breakpoint fusion did not appear to be mediated by a small homology region. Hence, even if the Alu elements in the 5′ breakpoint region was involved with the break, it did not seem to have mediated the incorrect repair. *IGHM* and *DCLRE1C* are characterized by a high content of transposable elements above the average of the human genomes, and this was associated with the high frequency of gross deletions (22). The *DOCK8* gene has a transposable element content of 37.6% (https://www.girinst.org/censor/). This content is pretty much the exact average of the whole human genome, which might predispose the vulnerability of gross deletions in the *DOCK8* gene. Alternatively, DOCK8 gene is located on chromosome 9q24 within a recombination hotspot that is characterized by subtelomeric repetitive sequences. Such locations might cause large intragenic germline deletions (23).

## Concluding Remarks

We presented a rare case of HIES. To the best of our knowledge, she was the first recognized and diagnosed case of DOCK8 deficiency in the Philippines. A high index of suspicion coupled with a good history and physical examination might lead the clinicians to the diagnosis of PID. Patients with HIES accompanied by warts and T-cell deficiency should be strongly suspected to have a DOCK8 deficiency. Newborn screening of TRECs can help identify such patients in infancy (24).

## Data Availability Statement

The datasets for this article are not publicly available due to concerns regarding participant/patient anonymity. Requests to access the datasets should be directed to the corresponding author.

## Ethics Statement

The studies involving human participants were reviewed and approved by the University of Santo Tomas Hospital (USTH) Research Ethics Committee. Written informed consent to participate in this study was provided by the participants' legal guardian/next of kin. Written informed consent was obtained from the individual(s) for the publication of any potentially identifiable images or data included in this article.

## Author Contributions

JV, RO, and AA provided clinical information. JV wrote the manuscript. K-WC performed the genetic analysis. Y-LL provided critical discussion. MZ performed bioinformatics analysis and edited the manuscript. HK supervised the study and edited the manuscript. All authors read and approved the final manuscript.

## Conflict of Interest

The authors declare that the research was conducted in the absence of any commercial or financial relationships that could be construed as a potential conflict of interest.
