# Supplementary material for: Hyper IgE Syndrome Associated With Warts: A First Case of Dedicator of Cytokinesis 8 Deficiency in the Philippines
Source: Front Pediatr. 2020 Oct 30;8:604725. doi: 10.3389/fped.2020.604725 (PMC7673426; doi:10.3389/fped.2020.604725)
Supplement: Supplementary file 1 [file Data_Sheet_1.docx]

Supplementary materials for “**Hyper IgE syndrome associated with warts: a first case of dedicator of cytokinesis 8 deficiency in the Philippines”** in Frontiers in Pediatrics

Jose Carlo Miguel M. Villanueva, Koon-Wing Chan, Remedios C. Ong, Agnes G. Andaya, Yu-Lung Lau, Menno C. van Zelm, and Hirokazu Kanegane

Corresponding author

Hirokazu Kanegane MD, PhD

Department of Child Health and Development, Graduate School of Medical and Dental Sciences, Tokyo Medical and Dental University, Tokyo, Japan

E-mail: hkanegane.ped@tmd.ac.jp

**Supplementary Table 1. Laboratory tests**

| Test | Patient | Unit | Reference value |
| --- | --- | --- | --- |
| Hemoglobin | 13.1 | g/dL | 13-14.5 |
| Hematocrit | 41 | % | 37-41 |
| Platelet count | 571 | x10^3^/μL | 150-350 |
| White blood cell count | 14,700 | /μL | 4,500-13,500 |
| Neutrophils | 64 | % |  |
|  | 9,408 | /μL | >1,500 |
| Lymphocytes | 19 | % |  |
|  | 2,793 | /μL | >1,500 |
| Eosinophils | 8 | % |  |
|  | 1,176 | /μL | <500 |
| Basophils | 1 | % |  |
| IgG | 1,841 | mg/dL | 650-1,600 |
| IgA | 181 | mg/dL | 40-350 |
| IgM | 10 | mg/dL | 50-350 |
| IgE | >5,000 | kU/L | <25 |
| TRECs | undetectable | copies/μgDNA | >10^2^ |
| KRECs | 2.0 x 10^2^ | copies/μgDNA | >10^2^ |

TRECs, T-cell receptor excision circles; KRECs, kappa-deleting recombination excision circles.

**Supplementary Table 2. National Institute of Health Scoring System for hyper IgE syndrome**

| Clinical findings | 0 | 1 | 2 | 3 | 4 | 5 | 6 | 7 | 8 | 10 |
| --- | --- | --- | --- | --- | --- | --- | --- | --- | --- | --- |
| Highest IgE (IU/mL) | <200 | 200-500 |  |  | 501-1,000 |  |  |  | 1,001-2,000 | >2,000 |
| Total skin abscesses/boils | None |  | 1-2 |  | 3-4 |  |  |  | >4 |  |
| Total pneumonias | None |  | 1 |  | 2 |  | 3 |  | >3 |  |
| Parenchymal lung abnormalities | None |  |  |  |  |  | Bronchiectasis |  | Pneumatocele |  |
| Other serious infection | None |  |  |  | Present |  |  |  |  |  |
| Fatal infection | None |  |  |  | Present |  |  |  |  |  |
| Highest eosinophils/μL | <700 |  |  | 701-800 |  |  | >800 |  |  |  |
| Newborn rash | None |  |  |  | Present |  |  |  |  |  |
| Eczema (worst stage) | None | Mild | Moderate |  | Severe |  |  |  |  |  |
| Sinusitis/otitis (in worst year) | 1-2 | 3 | 4-6 |  | >6 |  |  |  |  |  |
| Candidiasis | None | Oral, vaginal | Fingernail |  | Systemic |  |  |  |  |  |
| Retained primary teeth | None | 1 | 2 |  | 3 |  |  |  | >3 |  |
| Scoliosis (maximum curvature) | <10 |  | 10-14 |  | 15-20 |  |  |  | >20 |  |
| Minimal trauma fractures | None |  |  |  | 1-2 |  |  |  | >8 |  |
| Hyperextensibility | None |  |  |  | Present |  |  |  |  |  |
| Characteristic face | None |  | Mild |  |  | Present |  |  |  |  |
| Increased interalar distance | <1 SD | 1-2 SD |  | >2 SD |  |  |  |  |  |  |
| High palate | None |  | Present |  |  |  |  |  |  |  |
| Congenital anomaly | None |  |  |  |  | Present |  |  |  |  |
| Lymphoma | None |  |  |  | Present |  |  |  |  |  |

Red colors indicate clinical and laboratory features the patient fulfilled.

Score: <20: HIES unlikely; 20-40 HIES possible; >40: HIES probable.

Table lifted from the reference 13.

**Supplementary Table 3. DOCK8 score sheet**

| DOCK8 score  Mandatory: IgE> 10x normal range | | Feature | Scaled  points |
| --- | --- | --- | --- |
| A | Parenchymal lung abnormalities | No structural lung damage | 0.00 |
|  |  | Bronchiectasis | -30.00 |
|  |  | Pneumatoceles | -40.00 |
| B | Highest eosinophils/mL | <700 | 0.00 |
|  |  | 701-800 | 24.54 |
|  |  | >800 | 49.08 |
| C | Sinusitis, otitis (# episodes in wort year) | 1-2 | 0.00 |
|  |  | 3 | 15.50 |
|  |  | 4-6 | 31.00 |
|  |  | >6 | 62.00 |
| D | Retained primary teeth | None | 0.00 |
|  |  | 1 | -4.54 |
|  |  | 2 | -9.08 |
|  |  | 3 | -18.16 |
|  |  | >3 | -36.32 |
| D | Fractures with minor trauma | 0 | 0.00 |
|  |  | 1-2 | -36.36 |
|  |  | >2 | -72.72 |
|  | Total (Sum A-E) Scaled points | Cut off: >30 | 111.08 |

Red colors indicate clinical and laboratory features the patient fulfilled.

A score >30 predicts a high likelihood of a *DOCK8* mutation.

Table reproduced from the reference 8.


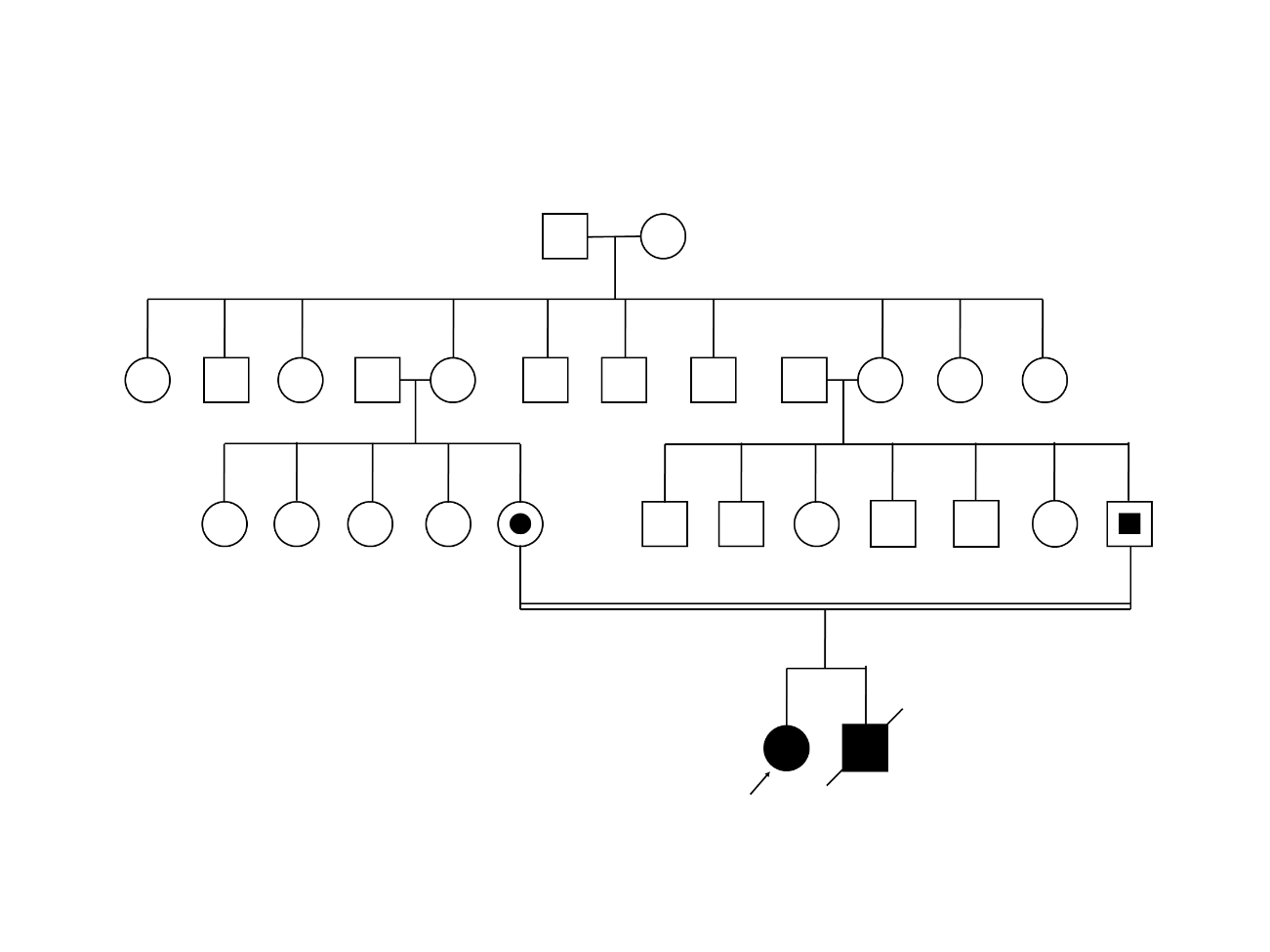


**Supplementary Figure 1. Genogram of the patient**

Arrow indicates the proband.


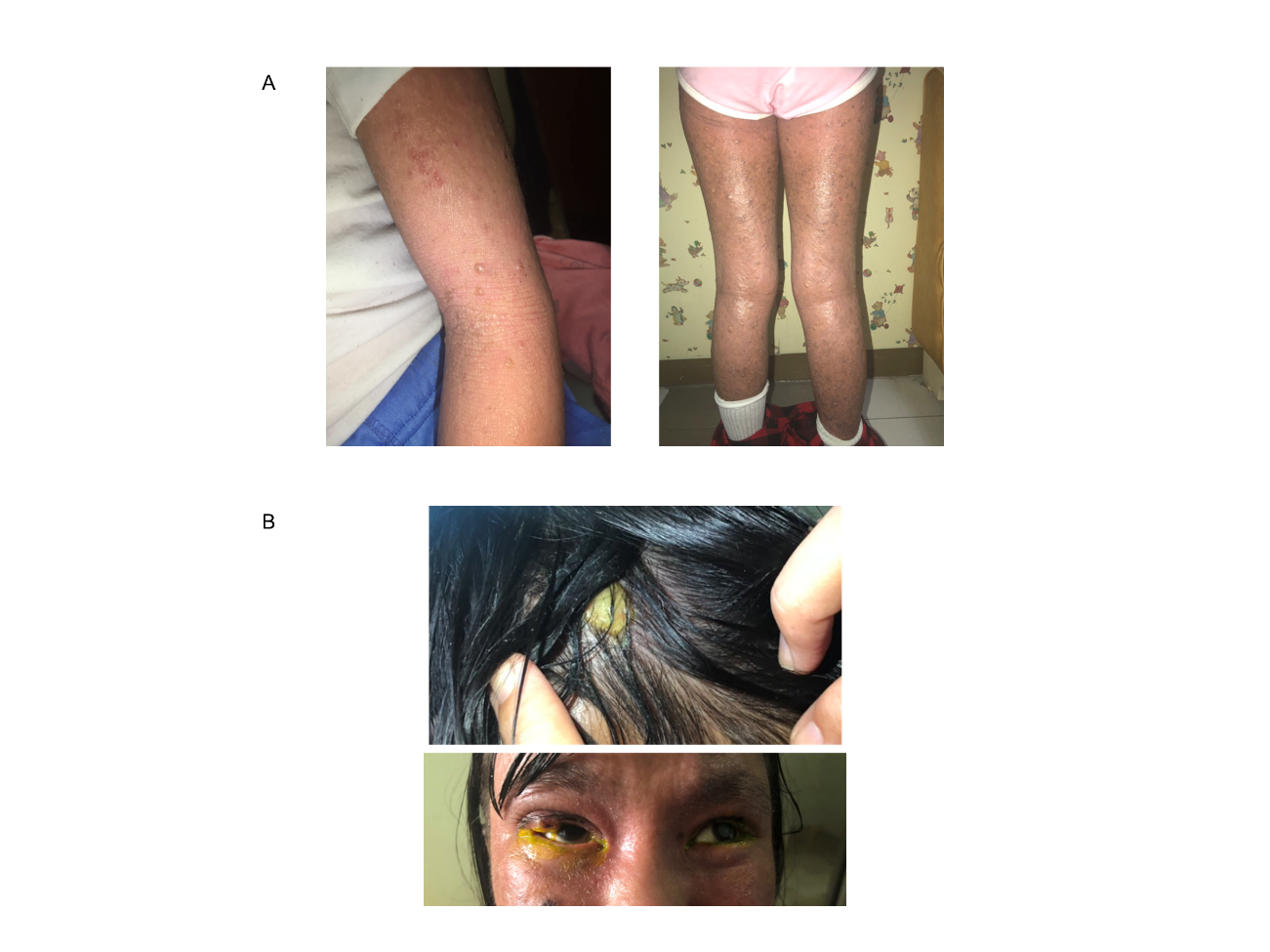


**Supplementary Figure 2. Clinical pictures of the patient**

1. Dry, lichenified skin with hyperlinearity, excoriations, and skin-colored papulonodular lesions on face, trunk, and extremities. (B) Abscess on the scalp, with yellowish discharge over the right eye, and an opacified left eye with absent red-orange reflex.


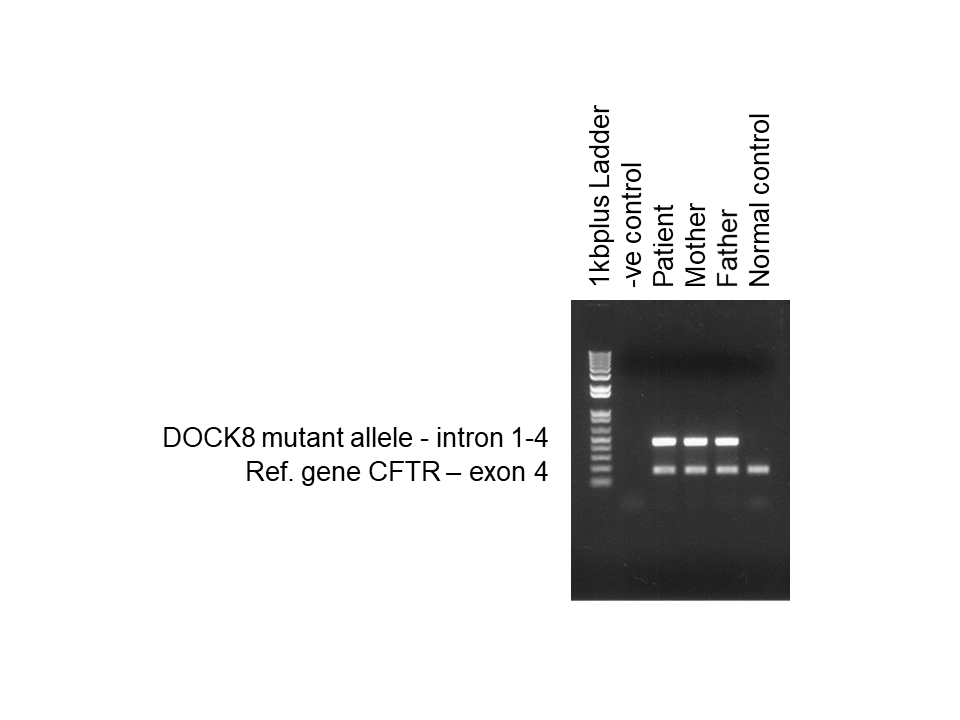


**Supplementary Figure 3. Parent carrier analysis of *DOCK8* gene**

Multiplex PCR was performed by co-amplification of *DOCK8* intron 1 to intron 4 (Primer pairs: *DOCK8*-Intron1FB and *DOCK8*-Intron4RC) with reference gene (*CFTR* exon 4). Electrophoresis of PCR products in 1.5% agarose gel indicated the present of mutant alleles in patient and parents but not in normal control.
